# Effect of Health Education on Dengue Fever: A Comparison of Knowledge, Attitude, and Practices in Public and Private High School Children of Jeddah

**DOI:** 10.7759/cureus.3809

**Published:** 2018-12-31

**Authors:** Hassan B Usman, Abdullah AlSahafi, Ola Abdulrashid, Najlaa Mandoura, Khalid Al Sharif, Adel Ibrahim, Leena Ahmed, Etidal Shamrani, Mona Shamia

**Affiliations:** 1 Epidemiology and Public Health, Directorate of Health Affairs for Public Health Division, Jeddah, SAU; 2 Family Medicine, Directorate of Health Affairs for Public Health Division, Jeddah, SAU; 3 Internal Medicine, Royal College of Surgeons, Dublin, IRL

**Keywords:** attitude, dengue fever, high schools, jeddah, knowledge, language, practice

## Abstract

Objective

More than half of the world’s population live in areas with a potential risk of acquiring dengue fever (DF). Health education interventions are effective, barring a language communication gap. The objective of this study was to estimate the effect of health education in the knowledge, attitude, and practices (KAP) towards DF control and prevention in public and private schools.

Materials and methods

We assessed the DF control and prevention strategy KAP of students of eight public and private schools in Jeddah, Saudi Arabia before the dengue health education intervention sessions (pre-I) and three months following the education intervention sessions (post-I) using the same closed-ended validated questionnaire. Schools and students were selected by a multistage stratified random sample method. Statistical analysis was done using the paired and independent T-test in IBM SPSS Statistics for Windows, Version 22.0 (IBM Corp., Armonk, NY).

Results

We found a significant mean difference in the overall knowledge (pre-I, 7.86 ± 2.61; post-I, 10.94 ± 2.35), attitude (pre-I, 5.16 ± 1.50; post-I 6.23 ± 1.30), and practice (pre-I, 2.96 ± 1.33; post-I, 3.94 ± 1.12) scores. Private schools scored better post-intervention scores in knowledge and practice compared to public schools in local and English language medium.

Conclusions

Health education programs are essential for DF prevention and management. Institutes whose populations consists of students with various language backgrounds should not be ignored. Bilingual educational sessions are important in such private institutes. Our results indicate additional emphasis is required on putting interventional knowledge into practice.

## Introduction

Dengue fever (DF) is an infectious disease transmitted mostly by the bite of the female Ades Aegypti mosquito and represents a great challenge to public health worldwide [1-2]. The expanding geographical distribution with high morbidity and mortality makes DF one of the more significant viral diseases [2]. In addition to DF vectors, human knowledge and behavior play an important role in the transmission of the disease [3]. According to the World Health Organization (WHO), around 2.5 billion people (two-fifths of the world’s population) are at risk of developing this disease. More than 50 million DF cases occur worldwide every year, and children are the most common victims of this deadly disease [2,4-5].

DF has received growing public health attention because of its high fatality rate ranging from 3% to 5% [6]. DF not only causes loss of human lives; each outbreak causes severe damage to the economy [6]. DF epidemics have been reported in many developing countries of sub-Saharan African and Asia [6]. DF has recently reemerged globally as one of the more important viral infections [7]. Unlike other diseases, DF occurs predominantly among urban populations. However, the risk of disease spread in the rural areas should also be considered [8].

The first case of DF was documented in Jeddah, Saudi Arabia in 1994. Since then, dengue virus surveillance was established which identified/confirmed the circulation of three dengue serotypes [9]. Millions of pilgrims transit through Jeddah, yielding a high risk for infectious diseases transmission. This puts both pilgrims and residents at high risk for developing DF [9-10]. DF incidence has increased significantly in Jeddah over the last few years with some confirmed recent outbreaks [10-11].

Considering dengue as a vector-borne disease, many initial attempts were directed at vector elimination only. These chemical vector control programs have limited feasibility mainly due to program maintenance costs [12]. As there is no available prophylactic vaccine for our region (the endemicity here is <50%) [7] and no specific treatment, vector control relies on the effective participation of all people in the area [13]. WHO and the Centers for Disease Control and Prevention recommend and emphasize community educational campaigns that highlight citizens’ responsibility in reducing vector breeding sites [5,14]. For this, a sustainable integrated approach is the key to prevent transmission by this vector. Intervention through health education programs plays an important role in promoting behavior change [4]. While electronic and print media’s contribution along with educational campaigns have increased awareness about DF, it remains unclear to what extent this knowledge is put into practice [15].

Health education should be culturally acceptable and offered in the local languages [16-17]. Some expatriate students studying in private schools have limited Arabic understanding [18]. In recent years, most dengue awareness sessions were arranged in the local language, leaving a gap in understanding of this important issue for these expatriate students [15,18]. Given that DF epidemiology is strongly associated with human habits and lifestyle, the evaluation of student knowledge, attitude, and practices (KAP) is of great importance [15]. With knowledge about the adverse effects and prevention, students can actively participate in control strategies for the dengue virus inside the home and their respective residential areas. Several studies have been conducted on DF at the regional and national levels in Saudi Arabia. However, comparison of public and private schools mainly focusing on the language in which awareness sessions are taught to school students (Arabic, English or both) and its effects have never been studied. This study was designed to assess and compare the improvement in DF KAP parameters for both public and private high school students.

## Materials and methods

The current study was conducted using a non-randomized design (i.e., before and after design) comparing public and private high schools in the four dengue prevalent districts of Jeddah, Saudi Arabia. Students in grade 10 to 12 in the selected schools were enrolled for this study. The target population was students in high schools as they can be good health educators for their parents, other family members, and society. The study duration was around eight months from February to October 2018. The implementation and evaluation of the health education program were done in three stages. The first stage was two to three weeks of pre-intervention (pre-I) questionnaire completion (i.e., pre-assessment). The second was three months for the delivery of health education on DF (the intervention) in small groups as these topics are usually not their curriculum part. The last was two to three weeks for completion of the post-intervention (post-I) questionnaire (i.e., post-assessment).

To estimate the current KAP regarding DF among high school students of the selected districts, we calculated the sample size using EpiTools online sample size calculator (http://epitools.ausvet.com.au/content.php?page=SampleSize). The mean knowledge score taken from other studies conducted in Jeddah [3,19] was 10 with a standard deviation of 4.5. Using these figures, keeping confidence interval (CI) at 95%, and the desired precision to be 0.5, the sample size was calculated. The calculated sample size was thus 312 students. This calculated sample size was then further enhanced by a percentage of 50% to accommodate for possible non-response/refusal/non-availability among the eligible study participants. Also sometimes it is difficult to get the data from the same students in the post-intervention session because of absenteeism. The overall sample size for the survey was thus proposed to be at least 500 participants across the selected schools. We took at least 75 students from each selected school. In the pre-assessment, we had a sample of 637 students, while some students were lost to follow-up on the day of post-intervention assessment. Therefore, a response rate of approximately 93% was noted.

The sample of schools and students was selected using the multistage stratified random sampling method. There are four dengue-prevalent districts/areas in Jeddah city according to the data taken from the infection control department of the Directorate of Health Affairs. Schools were stratified based on boys’ and girls’ schools irrespective of being governmental or private. From each district, we selected two schools in the district through a simple random sample selection approach. Three classes were selected from each school (one each from grades 10, 11, and 12), and we selected students within these classes through systematic sampling with a random start approach.

The questionnaire was developed after a careful review of the literature and translated into Arabic. The translation was performed by two bilingual professional translators who understand the content. The translated instrument was then back-translated into English by two other bilingual translators and compared to its original version. This procedure ensured clarity and comprehensibility of items. Any discrepancies in comparison were discussed, and minor adjustments were made. A team of trained researchers went to the schools, and, after taking informed consent, questionnaires were administered before the awareness sessions as a pre-test baseline. The same questionnaires were used three months later for the post-I evaluation. For expatriate students who did not understand Arabic, an English questionnaire was available. The same serial number (respondent ID) was allotted to the participants in pre-I and post-I evaluations.

In this intervention study design, DF health education sessions were imparted by our trained staff for three months. The health education was provided by school teachers, health educators, and nurses. Two volunteer teachers from each selected school were trained by the health education and infection control department of the Ministry of Health regarding DF’s symptoms, mosquito identification, and control measures. Simultaneously, several doctors and nurses also took awareness sessions in the selected schools. In the public schools, the sessions were given in Arabic, and in private schools, the sessions were given both in English and Arabic.

The educational sessions were carried out through lectures with the help of visual aids like flip charts and life cycle specimens followed by small group discussions. This gave the participants an opportunity to clarify their doubts about the problems related to DF and its management. At the end of health education sessions, each participant was asked to spread this educational message about DF control to family and friends. The main outcome variables were the effectiveness of health education via KAP scoring, and we compared pre-I KAP to post-I KAP scores

Data analysis was done using IBM SPSS Statistics for Windows, version 22.0 (IBM Corp., Armonk, NY). Overall mean scores were calculated and compared using the paired t-test. An independent t-test was used to compare public and private school outcomes. Scores were assigned to different variables in KAP; correct replies received a score of one, no/do not know replies received a score of zero. A total score of 17 was possible in the knowledge section, eight in the attitude section, and five in practices. Knowledge section included questions related to dengue symptoms, complications, mode of transmission and knowledge about the supportive environment. Attitude section included questions regarding their perception about the disease and practice section had questions regarding their daily practices like covering water tanks, preventing and disposal of stagnant water, window screening and use of repellants, etc. A score greater than the mean was considered a good score. Paired and independent t-test and chi-square tests were used to see the statistical difference, keeping 95% CI and p < 0.05 as statistically significant. Students of the selected school in grade 10 or above, present at the pre-assessment day and willing to participate were included in the study. Ethical approval was granted by the ethics committee of the Ministry of Health and Directorate of Health Affairs Jeddah (H-02-J-002-00847). Permission was also granted from the administration of each school.

## Results

Our study included 593 participants with a mean age of 16.2 ± 0.88 years. In our study population, 307 participants were boys (51.8%) and 286 were girls (48.2%). Most students (n = 403, 68%) were from Saudi. Students from both public (n = 327) and private (n = 266) schools were included in the study. Student’s grade wise distribution was as follows: Grade 10, 164 (27.7%); Grade 11, 141 (23.8%); Grade 12, 288 (48.6%). The most important sources of information about DF were school awareness campaigns/sessions (n = 435, 73.4%), TV/Radio (n = 397, 66.9%), and social media (n = 418, 70.5%). Students of public schools noted more dengue awareness sessions as compared to private schools (p = 0.038).

A paired t-test showed a significant overall improvement in KAP after the intervention. Pre-intervention KAP scores were 7.86 (± 2.61), 5.16 (± 1.50), and 2.96 (± 1.33), respectively, which improved to 10.94 (± 2.35), 6.23 (± 1.30), and 3.94 (± 1.12), respectively, post-intervention. An independent t-test was conducted to estimate the mean difference in the scores before and after intervention comparing public and private schools. The improvement in the post-intervention mean scores especially in the private schools was because of the inclusion of expatriate students. Most of the expatriate students gave their responses in English. This inclusion of expatriate students increased the number of correct responses in post-I which contributed to the overall increase in the mean scores. We noted a significant difference in the mean pre-intervention knowledge scores (p = 0.017), and public schools had better scores compared to private schools. However, following intervention, private schools had better knowledge scores (p = 0.038) as shown in Table 1. Similarly, we noted a significant difference in practice scores only after the health education intervention, and private school students scored better (p = 0.022).

**Table 1:** Independent t-test comparing public and private school scores (pre- and post-intervention; n = 593) (Total scores: Knowledge = 17, Attitude = 8, Practices = 5) CI, confidence interval; SD, standard deviation.

Variable		School		Mean difference	95 % CI		P value
		Public	Private		Lower	Upper	
		Mean (SD)	Mean (SD)				
Knowledge Scores	Pre-Intervention	8.09 (2.52)	7.57 (2.69)	0.516	0.094	0.938	0.017
	Post- intervention	10.76 (2.45)	11.16 (2.21)	-0.403	-0.785	-0.022	0.038
Attitude Scores	Pre- Intervention	5.18 (1.48)	5.12 (1.52)	0.065	-0.178	0.309	0.598
	Post- intervention	6.16 (1.40)	6.32 (1.15)	-0.158	-0.370	0.052	0.140
Practice Scores	Pre-Intervention	2.90 (1.34)	3.04 (1.32)	-0.146	-0.363	0.069	0.183
	Post- intervention	3.85 (1.22)	4.06 (.982)	-0.213	-0.395	-0.031	0.022

KAP correct responses of the main DF items by the study population before and after the intervention are presented in Table 2. Fever was correctly identified by students as major DF symptom (pre-I, 71%; post-I, 89%). However, other important DF symptoms which were included in the questionnaire like hemorrhage (pre-I, 35.5%), muscle and joint pain (pre-I, 47.7%), and skin rash (pre-I, 44.1%) were not recognized by most students even after the intervention.

**Table 2:** Main knowledge, attitude, and practices (KAP) questions and number of participants with the correct responses (n = 593) ^1^Transmission: through fly, tick/lice, all mosquito types, contact with other persons, blood transfusion. ^2^Environmental factors: active time of bite, presence in clean water, prefer indoor, can spread through a drop of water.

Knowledge variables		Correct answer (Percentage)	
		Pre-intervention	Post-intervention
Overall Knowledge about symptoms & complications (7 items)		279 (47.1)	381 (64.2)
Knowledge about main dengue symptoms	Fever	416 (70.2)	529 (89.2)
	Headache	309 (52.1)	471 (79.4)
	Muscle weakness	283 (47.7)	374 (63.1)
	Skin rash,	258 (43.5)	347 (58.5)
	Pain abdomen	234 (39.5)	326 (55.0)
	Pain and redness in eyes	239 (40.3)	313 (52.8)
Knowledge about important complication	Hemorrhage	212 (35.8)	307 (51.8)
Knowledge about mode of transmission and characteristics of vector (6 items)^1^		273 (46.1)	378 (63.8)
Knowledge about supportive environmental factors (4 items)^2^		267 (45.0)	368 (62.0)
Attitude variables			
I am afraid of dengue fever		394 (66.5)	483 (81.5)
Dengue is serious/dangerous illness		470 (79.2)	501 (84.5)
Dengue fever cannot be prevented		396 (66.7)	478 (80.6)
I am at risk/exposed to dengue		254 (42.8)	365 (61.6)
It is not necessary to seek immediate doctor consultation as there is no proper cure for it		364 (61.4)	451 (76.1)
There is no responsibility of public in dengue prevention		385 (64.9)	492 (83.0)
Elimination of breeding sites is a complete waste/unnecessary		369 (62.3)	431 (72.6)
There is a high chance of dengue spread in future		414 (69.8)	473 (79.7)
Practice variables			
Do you practice covering bottle & tanks		416 (70.2)	521 (87.8)
Do you practice stagnant water disposal at home & community		381 (64.2)	470 (79.2)
Do you use screening of window and doors at home		373 (62.9)	515 (86.8)
Do you use personal protection like repellants, nets, coils		240 (40.4)	383 (64.4)
Have you or municipality done fogging in your house		353 (59.6)	414 (69.8)

The independent t-test was performed to estimate the mean difference between boys and girls before and after health education intervention. We found no significant difference in the attitude and practice scores between the two groups before and after intervention as shown in Table 3. However, we noted a pre-intervention significant difference in the mean knowledge scores.

**Table 3:** Independent t-test comparing gender scores (pre- and post-intervention; n = 593) (Total scores: Knowledge = 17, Attitude = 8, Practices = 5)

Variable		Gender		Mean difference	95 % CI		P value
		Male	Female		Lower	Upper	
		Mean (SD)	Mean (SD)				
Knowledge Scores	Pre-Intervention	8.08 (2.68)	7.61 (2.51)	0.472	0.051	0.893	0.028
	Post-intervention	10.86 (2.30)	11.02 (2.41)	-0.158	-0.538	0.222	0.415
Attitude Scores	Pre-Intervention	5.23 (1.40)	5.08 (1.60)	0.154	-0.088	0.396	0.213
	Post-intervention	6.25 (1.20)	6.22 (1.40)	0.030	-0.180	0.240	0.778
Practice Scores	Pre-Intervention	2.95 (1.33)	2.98 (1.33)	-0.034	-0.250	0.180	0.751
	Post- intervention	3.93 (1.01)	3.96 (1.23)	-0.029	-0.212	0.152	0.747

## Discussion

The evaluation of student's KAP done in this study revealed much room for improvement. Major progress in the scores before and after the intervention highlights the need for continuous educational sessions based on recommended guidelines. In Saudi Arabia, recent DF outbreaks are a challenge to all health professionals and authorities [3,19]. Because of the increase in the incidence of DF cases, improving community knowledge and preventive practices regarding DF should be given due attention. There was no significant difference in the pre- and post-intervention attitude and practice scores in male and female students, and we found no disparity between the two groups—which addresses the region’s major misconception of considering girls as socially deprived [18]. A significant difference in pre-intervention knowledge score was observed, with boys being more knowledgeable than girl students. This finding, however, is not in agreement with the results reported by Al-Dubai [15] and Kamel [20]. This difference can be attributed to more male student exposure to outdoor activities, interaction with other people including health care staff, announcements in the mosques and more exposure to mass and social media.

More than 90% of the students were aware of the term “dengue” and identified it correctly as a disease, which was similar to results of studies conducted in India reporting 90% [21] and 83% [22], respectively. However, 78% of Brazilian students [23] and 67% of Thai students [24] knew about dengue, less than our study participants. A possible reason for the better knowledge score in our students could be the repeated health education campaigns targeting these schools. Study findings show mass media and social media including advertisement boards to be the most important sources of information for after-school health educational campaigns. These results match those observed in earlier studies by Acharya [21] and Shuaib et al. [25]. This suggests media in any form can play a vital role in disseminating dengue awareness messages and campaigns.

Surprisingly, the current study found low scores in all three domains of KAP (especially knowledge) before the intervention compared to countries where the DF has been endemic for decades [3,21-22]. Countries having DF for many years usually have more disease-related health educational and media campaigns [21-22]. As this disease is recently reemerged in Jeddah, a possible explanation for lesser scores can be less educational sessions. The overall knowledge score (post-intervention) was around 64%, nearly matching the 63% reported by Al-Zurfi et al. [26]. This score was, however, better than the 57% post-intervention score of a regional study [3]. The difference in knowledge between public and private school students can be attributed to two sessions at one time in private schools (one in Arabic and again in English). Studies prove that repetition of information helps in improved understanding [25-26]. Additionally, the inclusion of expatriate students in the overall analysis who understand the English language only may have increased the overall scores which were usually excluded from previous surveys. It is not a question on the effectiveness of training in Arabic language but a possible explanation could be the repeated pictorial messages using flip charts/ slides in Arabic and English helped in better understanding of concepts. Studies also prove bilingual facilitators help overcome language barriers, and in school health education sessions, ideally, the linguistic challenges can be clarified with a healthcare expert accompanied by a teacher [16-18]. This dual approach helps create a culturally appropriate and medically accurate description to use during health education sessions [18].

We noted an improvement in the post-intervention knowledge about mode of transmission (64%). However, the improvement we noted was much less than that reported by Bangkok [24] and Malaysian [26] studies (>80%). However, the results of our study were slightly better than the 60% score from a Brazilian study [23]. A high percentage of students still have the wrong perception about the mode of transmission and characteristics of the vector, indicating an opportunity for more focused education campaigns. 

Correct knowledge of signs, symptoms, and complications is important not only to recognize the disease but to seek appropriate and timely health care [19]. Like many regional [3,19] and international studies [15,20-23,26], fever was correctly identified by most study participants both pre- and post-intervention. However, symptoms like hemorrhage and skin rash were the least cited symptoms of DF which were consistent with findings of Shuaib, Al-Zurfi, and Farah et al. [25-27]. The knowledge that supportive environmental factors and eliminating the mosquito breeding sites would control the outbreak of DF improved to around 62%—much less than 89% of Malaysian students [26].

Regarding the attitude towards DF, most study participants were afraid and considered it a dangerous disease. However, it was interesting to note that most students in our study denied being at risk/exposed to the dengue virus. In contrast to this, Itrat et al. [28] reported that a majority Pakistanis considered themselves at risk of getting bitten by a dengue-carrying mosquito. This awareness may be attributed to deaths in the recent outbreaks and excessive media campaigns in Pakistan [27]. The overall attitude score of our study population was good (77.3%), similar to those of Malaysian students (79.9%) [26].

Post-intervention, the overall practice score was 77.4%, but, more encouraging, students covered bottles and tanks and disposed of stagnant water sources. This is a positive sign that students were taking actions against mosquito breeding sites. Our findings mirror those of the previous study conducted in Malaysia [26]. House fogging was reported in around 70% of participants, which is much more than what was observed in 34% of Malaysian students [26]. This high percentage is due to the government taking responsibility for spraying in private houses. Even though there was improvement reported in the use of personal protection against the dengue virus, the use of these should be further increased. Good knowledge does not necessarily lead to good practice. The present findings seem to be consistent with other research findings of a low incidence of use of personal protection [26-28].

The generalizability of these results is subject to certain limitations as the current study has only examined the KAP in dengue-prevalent districts where there has already been considerable focus on awareness programs.

## Conclusions

The present study was designed to determine the effect of health education intervention regarding DF in upper-grade school children. The evidence from this study highlights the need for bilingual health education programs (especially in institutes with a bilingual student population) about DF in improving knowledge with more emphasis on putting this knowledge into practice. Further improvement could be achieved by regular and more aggressive health education programs not only for the students but also for the wider community and in English where necessary. Social mobilization can also play its part in raising awareness and converting knowledge into practice for controlling DF epidemics in Jeddah.
